# Predictors of poor kidney function in patients with emphysematous pyelonephritis: a retrospective observational study

**DOI:** 10.1177/17562872261429824

**Published:** 2026-03-19

**Authors:** Anupam Choudhary, Kasi Viswanath Gali, Surag K.R., Anshuman Singh, Abhijit Shah, Krishnakanth A. V. B., Sunil Pillai, Padmaraj Hegde

**Affiliations:** Department of Urology, Kasturba Medical College, Manipal, Manipal Academy of Higher Education, Manipal, India; Department of Urology, Sri Venkateswara Institute of Medical Sciences, Tirupati, India; Department of Urology, Kasturba Medical College, Manipal Academy of Higher Education, Manipal, Karnataka 576104, India; Department of Uro-Oncology & Robotic Surgery, Chandan Cancer Institute, Chandan Hospital, Lucknow; Department of Urology, Kasturba Medical College, Manipal, Manipal Academy of Higher Education, Manipal, India; Department of Urology, Kasturba Medical College, Manipal, Manipal Academy of Higher Education, Manipal, India; Department of Urology, Kasturba Medical College, Manipal, Manipal Academy of Higher Education, Manipal, India; Department of Urology, Kasturba Medical College, Manipal, Manipal Academy of Higher Education, Manipal, India

**Keywords:** diabetes mellitus, drainage, kidney diseases, pyelonephritis, renal failure, renal insufficiency

## Abstract

**Background:**

Emphysematous pyelonephritis (EPN) is a well-known clinical condition characterized by an aggressive, gas-forming infection of the kidney caused by uropathogenic bacteria. There is growing interest in identifying the effects of EPN on long-term renal function and the factors that can help predict its impact.

**Objectives:**

This study evaluated risk factors for the development of poorly functioning kidneys (PFK) in patients diagnosed with EPN.

**Design:**

A retrospective study was conducted at a university teaching hospital on patients with EPN from January 2019 to December 2024.

**Methods:**

Data were collected from the prospectively maintained patient records, and patient demographics, comorbidities, clinical presentation, laboratory investigations, imaging characteristics, and interventions were analyzed. PFKs were defined as those with less than 15% differential function on radionuclide renography performed at follow-up. Data were analyzed using SPSS version 23 (IBM Corp.). A *p*-value <0.05 was considered statistically significant.

**Results:**

A total of 151 patients met the eligibility criteria. PFK was present in 23 patients (15.2%) on follow-up. Univariate analysis revealed random blood sugar (RBS) at presentation >200 mg/dL (*p* < 0.0001), >50% parenchymal involvement on imaging (*p* < 0.00001), Huang-Tseng Class 3b (*p* < 0.00001), and persistence of gas in the renal parenchyma on follow-up imaging (*p* < 0.0001) to be significantly associated with the development of PFK. Multivariate Analysis revealed that >50% parenchymal involvement on initial imaging was an independent and significant predictor of PFK.

**Conclusion:**

EPN is a fulminant renal infection with a high risk of renal functional deterioration. Renal parenchymal involvement of >50% on initial imaging emerged as the most significant and independent predictor of poor renal function. Additional contributory factors included RBS >200 mg/dL, Huang-Tseng Class 3b, and persistence of gas in the renal parenchyma on follow-up. Recognizing these clinical and radiological predictors can support early risk stratification, guide patient counseling, and inform individualized management strategies aimed at preserving renal function.

## Plain language summary

Poorly functioning kidneys in patients with severe infection of kidneys

Emphysematous pyelonephritis (EPN) is a dangerous gas-forming kidney infection, often linked to diabetes, that can cause long-term kidney damage. In a study of 151 patients, 15% ended up with poorly functioning or nonfunctioning kidneys. The strongest predictor of poor outcome was more than 50% of the kidney being affected by gas on the first computed tomography (CT) scan. Other risk factors included high blood sugar at diagnosis (>200 mg/dL), severe disease on CT (Huang-Tseng Class 3b), and gas persisting in the kidney on follow-up imaging. Recognizing these signs early can help guide treatment and improve long-term kidney health.

## Introduction

Emphysematous pyelonephritis (EPN) is a well-recognized, aggressive necrotizing infection of the kidney caused by gas-forming uropathogens. The condition is potentially fatal if not diagnosed and treated promptly. Historically, EPN was associated with mortality rates exceeding 70%; however, advances in imaging, broad-spectrum antibiotics, critical-care support, and minimally invasive drainage procedures have reduced mortality to approximately 20%—25%.^1^ The paradigm of management has also shifted toward organ-preserving approaches, with emergency nephrectomy or open incision and drainage (I&D) now reserved for select, refractory cases.^2^

Most previous studies on EPN have primarily focused on identifying predictors of mortality and the need for nephrectomy. In contrast, relatively little attention has been paid to the long-term renal functional outcomes among survivors. Preservation of renal function is increasingly recognized as a key determinant of quality of life, particularly because many patients with EPN have pre-existing comorbidities such as diabetes mellitus, hypertension, or obstructive uropathy that predispose them to chronic kidney disease and progressive renal impairment. In addition, renal recovery following severe infection is often unpredictable, and subtle functional loss may progress silently over time despite apparent clinical resolution. Understanding the factors contributing to renal dysfunction is therefore crucial for optimizing follow-up, guiding intervention, and counseling patients on prognosis.

Recent literature suggests that several clinical, biochemical, and radiological factors may influence renal recovery following EPN or severe pyelonephritis. These include the extent of renal parenchymal destruction on imaging, the Huang-Tseng classification grade, glycemic control at presentation, and the persistence of infection or gas on follow-up scans.^3,4^ High random blood sugar (RBS) levels, poor metabolic control, and extensive parenchymal involvement have been linked to irreversible renal damage due to ischemia, cortical necrosis, and diabetic microangiopathy.^3,5^ Additionally, delayed drainage or inadequate infection control may further compromise renal function despite initial clinical improvement. However, despite improvements in diagnosis and management, data focusing specifically on predictors of long-term renal function following EPN remain scarce, particularly from large, systematically analyzed cohorts in the current era of minimally invasive therapy.

In recent years, there has been a paradigm shift in the management of EPN from immediate nephrectomy to a more conservative, renal-preserving approach guided by advances in imaging, percutaneous drainage (PCD), and intensive care.^6^ This evolution has been supported by evidence demonstrating that many patients can recover without the need for surgical removal of the affected kidney, provided timely drainage and antibiotic therapy are instituted. Our institution has adopted a stepwise, organ-preserving treatment strategy wherever feasible, reserving nephrectomy for refractory or life-threatening cases. Within this framework, the present study focuses specifically on patients managed conservatively, aiming to identify predictors of renal functional decline among those with preserved kidneys.

Our study aimed to evaluate risk factors for the development of poorly functioning kidney (PFK) in patients diagnosed with EPN.

## Methods

A retrospective observational study was conducted at a university teaching hospital among patients diagnosed with EPN from January 2019 to December 2024. Inclusion criteria consisted of adults aged ⩾18 years with a confirmed diagnosis of EPN on computed tomography (CT) scans and with adequate follow-up data for assessment of post-treatment renal function. Exclusion criteria included patients with pre-existing chronic kidney disease, those lost to follow-up, and those requiring emergency nephrectomy. As this was a retrospective analysis based on hospital records, mortality data beyond the initial hospitalization were not consistently available; therefore, only patients with complete follow-up and post-treatment renal function assessment were included.

Data were collected from prospectively maintained patient records. Data collected included patient demographics, comorbidities, clinical presentation (such as hemodynamic instability and ICU stay), laboratory parameters (including RBS and renal function tests), and interventions such as double J (DJ) stenting, percutaneous nephrostomy (PCN) or PCD, and I&D. Imaging characteristics were evaluated using non-contrast CT (NCCT) scans and classified according to the Huang-Tseng staging system. Additional imaging variables assessed included renal parenchymal involvement, categorized as <25%, 25%—50%, 50–75%, or >75% based on the extent of renal parenchyma involved by gas on the maximal longitudinal coronal section and the presence of obstruction or obstructive calculi. Urinary tract obstruction was defined as the presence of hydronephrosis with or without obstructive calculi on NCCT scan. Follow-up CT imaging was performed based on clinical indication, typically 14–21 days after the initial CT scan, or earlier in patients with persistent symptoms or inadequate clinical response. Persistence of gas was defined as the continued presence of intrarenal or perinephric gas on follow-up imaging compared with baseline. PFK was defined as those with less than 15% differential function on radionuclide renography performed at least 4 weeks after the initial presentation. Follow-up data were obtained through electronic medical records to evaluate renal function outcomes over time.

## Statistical analysis

Data were entered and analyzed using SPSS version 23 (IBM Corp., USA). Descriptive statistics were expressed as mean ± standard deviation (SD) for continuous variables and as frequencies (percentages) for categorical variables. The normality of continuous variables was assessed using the Shapiro–Wilk test. Associations between categorical variables were evaluated using the Chi-square or Fisher’s exact test as appropriate, while continuous variables were compared using the independent sample *t* test.

Univariate analysis was performed to identify variables associated with PFK. Variables showing statistical significance on univariate analysis were subsequently entered into a binary logistic regression model to determine independent predictors of PFK, and odds ratios (OR) with 95% confidence intervals (CI) were calculated. A *p* value <0.05 was considered statistically significant.

To minimize instability from sparse subgroup data, variables with small cell counts were consolidated into broader, clinically relevant categories before analysis.

The reporting of this study conforms to the Strengthening the Reporting of Observational Studies in Epidemiology (STROBE) statement.^7^ The STROBE checklist is found in Supplemental Material 1.

## Results

A total of 151 patients met the inclusion and exclusion criteria and were included in the study. The mean (SD) age of the study population was 57.2 ± 12.2 years. Of these, 86 (56.9%) were male, and 65 (43.1%) were female. Diabetes mellitus was present in 140 patients (92.7%), while 70 patients (46.3%) had hypertension. Patient demographics, clinical presentation, laboratory findings, and imaging characteristics are summarized in Table 1.

**Table 1.** Patient demographics, clinical presentation, and imaging characteristics.

Variable	Frequency (*n*)	Mean ± SD
**Patient demographics**		
Age (years)		57.2 ± 12.2
Male	86 (56.9%)	
Female	65 (43.1%)	
Diabetes mellitus	140 (92.7%)	
Hypertension	70 (46.3%)	
**Clinical characteristics at presentation**		
HD instability	46 (30.5%)	
ICU stay	55 (36.4%)	
Need for RRT	12 (7.9%)	
Presence of AKI	89 (58.9%)	
**Laboratory investigations**		
RBS in mg/dL at presentation		288.4 ± 144.1
Serum creatinine (mg/dL) at presentation		2.1 ± 1.5
Serum creatinine at discharge (mg/dL)		1.5 ± 1.1
Positive urine culture	75 (49.7%)	
Positive blood culture	56 (37.1%)	
**Imaging characteristics (on NCCT)**		
**Huang-Tseng stage**		
Huang-Tseng 1	74 (49%)	
Huang-Tseng 2	33 (21.9%)	
Huang-Tseng 3a	21 (13.9%)	
Huang-Tseng 3b	23 (15.5%)	
**Percentage of parenchymal involvement (affected side)**		
<25%	44 (29.1%)	
**25**%—**50%**	**11 (7.3%)**	
** 50**%—**75%**	**7 (4.6%)**	
** >75%**	**15 (9.9%)**	
** NA (Huang-Tseng 1)**	**74 (49%)**	
**Presence of obstruction (affected side)**		
No obstruction	119 (78.8%)	
Mild hydronephrosis	19 (12.6%)	
Moderate hydronephrosis	11 (7.3%)	
Gross Hydronephrosis	2 (1.3%)	
**Presence of obstructive calculi (affected side)**		
Obstructive calculi	24 (15.9%)	

AKI, acute kidney injury; HD, Hemodynamic; ICU, intensive care unit; NCCT, non-contrast computed tomography; RBS, random blood sugar; RRT, renal replacement therapy.

Hemodynamic instability at presentation was noted in 46 patients (30.5%); ICU admission was required in 55 patients (36.4%); and 12 patients (7.9%) required renal replacement therapy. Acute kidney injury at presentation was observed in 89 patients (58.9%).

**Figure 1.**
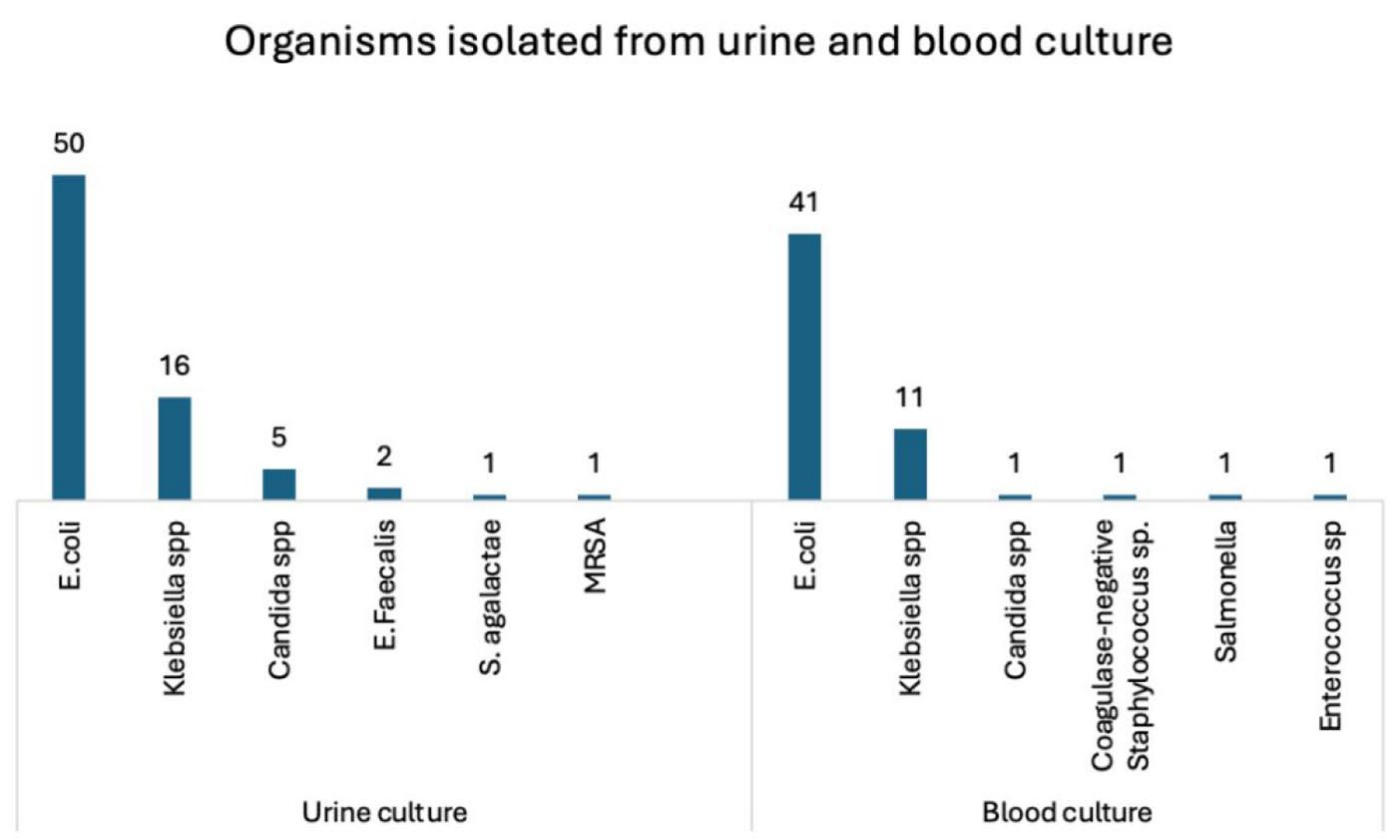
Bacterial isolates from urine and blood cultures.

The mean (SD) RBS at presentation was 288.4 ± 144.1 mg/dL. Mean (SD) serum creatinine at presentation was 2.1 ± 1.5 mg/dL, which decreased to 1.5 ± 1.1 mg/dL at discharge. A positive urine culture was noted in 75 patients (49.7%), and blood cultures were positive in 56 patients (37.1%). The microbiological profile is depicted in Figure 1, with *Escherichia coli* being the most frequently isolated organism.

Based on the classification of findings on NCCT scan, 74 patients (49%) were categorized as Huang-Tseng Class 1, 33 (21.9%) as Class 2, 21 (13.9%) as Class 3a, and 23 (15.5%) as Class 3b. The percentage of renal parenchymal involvement on the affected side was <25% in 44 patients (29.1%), 25%—50% in 11 (7.3%), 50%—75% in 7 (4.6%), and >75% in 15 patients (9.9%). Hydronephrosis was observed in 32 patients (22.2%), while obstructive calculi were noted in 24 patients (15.9%). Hydronephrosis was included in the univariate analysis; however, it did not show a statistically significant association with PFK development and was therefore not retained in the multivariate regression model.

Interventions required and follow-up findings are summarized in Table 2. Among the cohort, 77 patients (50.9%) underwent DJ stenting, 15 (9.9%) required PCN, 10 (6.6%) underwent PCD, and 3 patients (1.9%) required I&D, in addition to intravenous antibiotics and supportive care. A total of 46 patients (29.5%) were managed conservatively with intravenous antibiotics and supportive therapy alone.

**Table 2.** Intervention and follow-up findings.

Treatment received	Stage 1	Stage 2	Stage 3a	Stage 3b	Total
Double J stenting	36 (23.8%)	18 (11.9%)	12 (7.9%)	11 (7.3%)	77 (50.9%)
PCN	1 (0.7%)	5 (3.3%)	4 (2.6%)	5 (3.3%)	15 (9.9%)
PCD	0	3 (1.9%)	3 (1.9%)	4 (2.6%)	10 (6.6%)
I&D	0	1 (0.7%)	1 (0.7%)	1 (0.7%)	3 (1.9%)
Follow-up					
Serum creatinine (mg/dL) at follow-up		Mean ± SD		1.4 ± 1.04	
Repeat CT findings: EPN persistent		Yes		20 (13.2%)	
NFK/PFK		Yes		23 (15.2%)	

CT, computed tomography; EPN, emphysematous pyelonephritis; I&D, incision and drainage; NFK, nonfunctioning kidney; PCD, percutaneous drainage; PCN, percutaneous nephrostomy; PFK, poorly functioning kidney.

At follow-up, the mean (SD) serum creatinine was 1.4 ± 1.04 mg/dL. Persistent gas on follow-up CT imaging was observed in 20 patients (13.2%). A radionuclide renogram was performed in patients with clinical or radiological suspicion of NFK/PFK, which confirmed PFK/NFK in 23 patients (15.2%) of the study cohort.

Univariate analysis of factors associated with the development of a NFK/PFK following EPN revealed RBS at presentation >200 mg/dL (*p* < 0.0001), >50% parenchymal involvement on initial imaging (*p* < 0.00001), Huang-Tseng classification Class 3b (*p* < 0.00001), and persistence of gas in the renal parenchyma on follow-up imaging (*p* < 0.0001) to be significantly associated with the development of PFKs on Univariate Analysis. The association of other clinical, laboratory, and imaging parameters with renal functional outcomes is summarized in Table 3. Variables significant on univariate analysis were included in the multivariate binary logistic regression model. After adjustment, only >50% parenchymal involvement on initial imaging remained an independent predictor of PFK (adjusted OR 648.2, 95% CI: 35.6–11,560, *p* < 0.001; Table 4).

**Table 3.** Univariate analysis of various parameters with NFK/PFK.

Parameters	NFK/PFK		*p* Value	Odds ratio (95% CI)
	Yes (*n* = 23)	No (*n* = 128)		
Age <50 years	7	34	0.7	1.2 (0.45–3.19)
Age ⩾50 years	16	94		
Male	7	58	0.18	0.5 (0.2–1.3)
Female	16	70		
Diabetes (Yes)	23	117	0.2	4.6 (0.2–80.9)
Diabetes (No)	0	11		
Hypertension (Yes)	11	58	0.8	1.1 (0.4–2.69)
Hypertension (No)	12	70		
Need for RRT (Yes)	1	11	0.4	0.49 (0.05–3.9)
Need for RRT (No)	22	117		
RBS at presentation <200 mg/dL	3	42	<0.0001*	2.5 (0.7–9.1)
RBS at presentation >200 mg/dL	20	109		
Creatinine at presentation (>2 mg/dL)	9	51	0.9	0.9 (0.3–2.4)
Creatinine at presentation (<2 mg/dL)	14	77		
Hydronephrosis (Yes)	6	26	0.5	1.2 (0.4–3.3)
Hydronephrosis (No)	17	102		
Parenchymal involvement <25%	2	116	<0.00001*	
Parenchymal involvement 25%—50%	1	10		
Parenchymal involvement 50%—75%	6	1		
Parenchymal involvement >75%	14	1		
Huang-Tseng 1	1	73	<0.00001*	
Huang-Tseng 2	5	28		
Huang-Tseng 3a	4	17		
Huang-Tseng 3b	13	10		
Repeat CT findings: EPN persistent (Yes)	10	10	<0.0001*	9.1 (3.2–25.8)
Repeat CT findings: EPN persistent (No)	13	118		

CT, computed tomography; EPN, emphysematous pyelonephritis; NFK, nonfunctioning kidney; PFK, Poorly functioning kidney; RBS, random blood sugar; RR, renal replacement therapy; RRT, renal replacement therapy.

**Table 4.** Multivariate analysis of various parameters with NFK/PFK.

Predictor	Odds ratio	95% CI	*p* Value
RBS at presentation >200 mg/dL	1.00	0.99–1.01	0.26
Parenchymal involvement >50%	648.2	35.6–11,560	<0.001
Huang-Tseng 3b	0.54	0.01–11.5	0.72
Repeat CT findings: EPN persistent (Yes)	1.44	0.07–48.1	0.82

Variables significant on univariate analysis were entered into multivariate binary logistic regression. Categories with sparse data (parenchymal involvement 50%—75% and >75%) were combined into a single >50% category to ensure model stability.

CT, computed tomography; NFK, nonfunctioning kidney; PFK, Poorly functioning kidney; RBS, random blood sugar.

## Discussion

EPN is a severe, necrotizing infection of the kidney caused by gas-forming organisms and remains a urological emergency requiring timely diagnosis and intervention.^8^ Consistent with established literature, diabetes mellitus and obstructive uropathy were the predominant risk factors in our cohort, with over 90% of patients having diabetes and 15.9% having obstructive calculi.^9,10^ Poor glycemic control promotes bacterial proliferation, impairs host immunity, and contributes to microvascular ischemia, thereby increasing susceptibility to severe renal infection.^5^ Obstructive uropathy further exacerbates disease severity by causing urinary stasis, serving as a nidus for bacterial colonization—particularly in the presence of calculi—and facilitating ascending infection.^9,10^ In our cohort, hydronephrosis was observed in 22.2% of patients. However, hydronephrosis was not independently associated with the development of PFK, possibly because obstruction was relieved early through timely decompression with DJ stenting or PCN insertion. The mean age of affected patients was 57 years, similar to previous reports indicating that EPN predominantly affects older individuals with multiple comorbiditie.^11,12^ These underlying risk factors not only predispose patients to EPN but may also influence subsequent renal recovery, underscoring the importance of identifying prognostic indicators of long-term renal functional outcomes.

Our findings shift the focus from mortality to long-term renal functional outcomes, which remain underexplored in the literature. While previous studies have identified predictors of mortality, such as shock, thrombocytopenia, and altered sensorium, there is limited understanding of factors predicting renal function deterioration in survivors of EPN.^4,13^ Our study addresses this gap by evaluating clinical and radiological parameters that influence renal recovery after infection resolution. This change in focus has important clinical implications, as many EPN survivors continue to experience varying degrees of renal impairment despite apparent infection control. Identifying modifiable predictors early in the disease course could enable more personalized management strategies, including aggressive infection control, tighter glycemic control, and structured follow-up imaging to monitor functional recovery.

Our study identified *E. coli* as the most common causative organism, followed by Klebsiella species, Candida, and others. These findings are consistent with prior studies.^4,12^ Although microbiological patterns influence disease severity, our analysis indicates that host factors and imaging characteristics are more strongly correlated with long-term renal outcomes. This suggests that the host response, metabolic milieu, and extent of parenchymal destruction may play a more decisive role in determining renal prognosis than the specific pathogen involved.

EPN is best diagnosed using a CT scan, which remains the imaging modality of choice. While the Huang-Tseng classification is well established for assessing disease severity, its correlation with long-term renal function is less defined.^4^ In our study, most patients had less severe disease (Types 1 and 2), explaining the absence of mortality. However, within this relatively favorable spectrum, renal outcome varied markedly, emphasizing that radiological extent—not only class—drives functional prognosis.

Among all variables analyzed, >50% renal parenchymal involvement on CT scan emerged as the most significant and independent predictor of PFK. This observation suggests that extensive gas formation and parenchymal destruction reflect irreversible structural injury, resulting in persistent functional loss. Similar findings were reported by Kapoor et al., who linked >50% involvement to delayed nephrectomy, supporting the concept that greater parenchymal involvement correlates with poorer renal preservation.^3^

Other significant predictors in our cohort included RBS >200 mg/dL, Huang-Tseng Class 3b, and persistence of parenchymal gas on follow-up imaging. The association between elevated RBS and poor renal function likely reflects pre-existing diabetic nephropathy compounded by acute ischemic and infectious insult. Class 3b disease indicates extensive parenchymal and perinephric spread, signifying severe tissue destruction. Persistence of gas at follow-up represents ongoing infection or non-viable parenchyma, both markers of irreversible renal damage. Collectively, these factors highlight that both metabolic control and radiological extent must be considered together when predicting renal outcomes in EPN. Taken together, these findings underscore that radiological severity, glycemic control, and persistent infection are critical determinants of renal recovery following EPN. Early identification of these risk factors can also help stratify patients for follow-up renography, long-term nephrology care, or preventive interventions aimed at renal preservation.

Management of EPN has evolved from routine emergency nephrectomy to a more conservative, renal-preserving approach guided by patient stability, imaging findings, and response to drainage and antibiotics. Early nephrectomy, once the standard of care due to high mortality rates, has largely been replaced by broad-spectrum antibiotics, strict glycemic control, and image-guided drainage procedures such as percutaneous nephrostomy (PCN) or percutaneous drainage (PCD).^6^ Nephrectomy is now reserved for patients with uncontrolled sepsis despite adequate drainage, extensive bilateral disease, or non-viable kidneys on imaging. At the same time, conservative treatment is preferred in hemodynamically stable patients with localized disease and adequate drainage access. Our findings complement this evolving paradigm by identifying imaging and biochemical parameters that may predict renal functional decline even in patients who recover without nephrectomy, thereby assisting clinicians in risk stratification and long-term follow-up planning.

Based on our findings and current evidence, we propose a simplified clinical framework for managing EPN. Patients presenting with hemodynamic instability or >50% parenchymal involvement on CT scan should be considered high risk and evaluated for early image-guided drainage or timely nephrectomy if unresponsive to initial therapy. In contrast, patients with ⩽50% involvement and stable clinical status can often be managed conservatively with antibiotics, glycemic control, and close renal function monitoring. Recognition of predictors such as poor glycemic control, Huang-Tseng Class 3b disease, and persistent parenchymal gas allows early identification of patients at risk for developing a PFK, enabling individualized follow-up and timely intervention.

Our study has certain limitations. The retrospective, single-center design limits generalizability, and external validation is required. Patients undergoing nephrectomy were excluded to maintain homogeneity of renal outcome analysis; thus, our findings may underestimate total renal loss. Additionally, because treatment modality (e.g., DJ stenting, PCN, or PCD) was determined by clinical judgment rather than random assignment, the potential for selection bias cannot be excluded. Therefore, intervention type was not used as a predictor variable in regression analysis to minimize this bias. Another limitation of our study is the absence of an a priori sample size calculation, as the retrospective design required inclusion of all available eligible cases rather than a predetermined sample size. Nonetheless, the results highlight important associations that warrant further prospective, multicentric validation to strengthen predictive accuracy and guide individualized patient counseling.

## Conclusion

EPN is an acute, fulminant renal infection with a high risk of renal functional deterioration, potentially leading to poorly functioning or nonfunctioning kidneys. In our study, >50% parenchymal involvement on initial imaging emerged as the most significant and independent predictor of poor renal function. Additional contributory factors included RBS at presentation >200 mg/dL, Huang-Tseng classification Class 3b, and persistence of gas in the renal parenchyma on follow-up imaging. Recognizing these clinical and radiological predictors can support early risk stratification, guide patient counseling, and inform individualized management strategies aimed at preserving renal function.

## Supplemental Material

sj-docx-1-tau-10.1177_17562872261429824 – Supplemental material for Predictors of poor kidney function in patients with emphysematous pyelonephritis: a retrospective observational study Supplemental material, sj-docx-1-tau-10.1177_17562872261429824 for Predictors of poor kidney function in patients with emphysematous pyelonephritis: a retrospective observational study by Anupam Choudhary, Kasi Viswanath Gali, Surag K.R., Anshuman Singh, Abhijit Shah, Krishnakanth A. V. B., Sunil Pillai and Padmaraj Hegde in Therapeutic Advances in Urology

## Declarations

**Ethics approval and consent to participate:** Ethical approval for the study was obtained from the institutional ethics committee of Kasturba Medical College and Kasturba Hospital (IEC1-29-2025). The study was conducted adhering to the Declaration of Helsinki. A waiver of informed consent was obtained, given the retrospective nature of the study.

**Consent for publication:** Not applicable.

**Availability of data and materials:** Not applicable.
